# The effect of context and audio-visual modality on emotions elicited by a musical performance

**DOI:** 10.1177/0305735616670496

**Published:** 2016-10-26

**Authors:** Eduardo Coutinho, Klaus R. Scherer

**Affiliations:** 1 Department of Music, University of Liverpool, UK; 2 University of Geneva, Switzerland and University of Munich, Germany

**Keywords:** contextual factors, emotion, laboratory, live performance, music

## Abstract

In this work, we compared emotions induced by the same performance of Schubert Lieder during a live concert and in a laboratory viewing/listening setting to determine the extent to which laboratory research on affective reactions to music approximates real listening conditions in dedicated performances. We measured emotions experienced by volunteer members of an audience that attended a Lieder recital in a church (Context 1) and emotional reactions to an audio-video-recording of the same performance in a university lecture hall (Context 2). Three groups of participants were exposed to three presentation versions in Context 2: (1) an audio-visual recording, (2) an audio-only recording, and (3) a video-only recording. Participants achieved statistically higher levels of emotional convergence in the live performance than in the laboratory context, and the experience of particular emotions was determined by complex interactions between auditory and visual cues in the performance. This study demonstrates the contribution of the performance setting and the performers’ appearance and nonverbal expression to emotion induction by music, encouraging further systematic research into the factors involved.

The process of emotion induction through music has proven to be a major challenge to empirical research in different disciplines. One reason for this might be that although the emotions expressed in a piece of music tend to be based on a combination of acoustic and musical-structural features (Gabrielsson & Lindström, 2010), the emotions experienced by listeners are also influenced by a variety of parameters related to listener traits and states, musicians’ performance, and listening and cultural contexts (e.g., Gabrielsson, 2002; Scherer & Zentner, 2001; Scherer, Zentner, & Schacht, 2001–2002). Therefore, a comprehensive empirical investigation of emotional experiences during exposure to music must consider a wide range of variables in addition to the music itself if investigators are to achieve a broader understanding of the emotional power of music (see also McPherson & Schubert, 2004).

In a recent attempt to categorise and operationalise such issues, Scherer and Coutinho (2013) presented an integrated framework that allows a description of the nature and substrate of a wide range of emotional experiences induced by music, considering a variety of possible modulatory effects. In particular, the authors elaborated on the implications of three main groups of factors related to the listening context to the process of emotion induction—performance, listener (or individual), and contextual factors—that may, directly or indirectly, have an influence on the emotions produced by music in a particular listener or group of listeners. *Performance factors* include at least two different (albeit linked) dimensions. The first relates directly to the auditory experience and to the way in which a piece of music is executed by singers and/or instrumentalists. This is an extensively studied field, and it is well known that cues such as tempo, dynamics, timing, timbre, and articulation are among the most important acoustic building blocks used by performers to achieve emotional expression (e.g., Juslin & Timmers, 2010). The second factor concerns domains outside the auditory experience and refers to the effects of iconic, indexical, and symbolic information communicated during the performance (Dowling & Harwood, 1986; Peirce, 1931–1935, 1958), such as the stable identity of the performer (e.g., physical appearance, expression, and reputation), the performer’s technical and interpretative skills, transient performance-related variables (e.g., interpretation, concentration, motivation, mood), and performance manners (body movements, gestures, stage presence, audience contact, etc.). As examples of the importance of these factors, Thompson, Graham, and Russo (2005) have shown that visual aspects of performance (facial expressions and bodily movements) reliably influence affective interpretations of music. Vines, Krumhansl, Wanderley, Dalca, and Levitin (2011) presented strong evidence that the performers’ stage behaviours (in terms of expressivity) make unique contributions to the communication of emotion to the audience. Furthermore, various authors have also demonstrated the importance of the performers’ attractiveness and attire (e.g., Howard, 2012; Wapnick, Mazza, & Darrow, 1998).

*Listener-related factors* pertain to the characteristics of an individual, but also to the socio-cultural identity of the listener and the symbolic musical coding convention prevalent in a particular culture or subculture. These factors can be summarised as stable dispositions, transient listener states, and musical expertise. Stable dispositions include individual differences in age (e.g., motivational and selective neuropsychological decline; see, for instance, Vieillard, Didierjean, & Maquestiaux, 2012) and gender (e.g., Nater, Abbruzzese, Krebs, & Ehlert, 2006); in memory (including learned associations and conditioning; see Jäncke, 2008); and in inference dispositions based on personality (e.g., Rusting & Larsen, 1997), socio-cultural factors (e.g., Basabe et al., 2000; Egermann et al., 2011), prior experiences (e.g., Bigand & Poulin-Charronnat, 2006), among other things. Transient listener states such as motivational state, concentration, or mood may also affect emotional inference from music (cf. Cantor & Zillmann, 1973). Musical expertise includes those musical capacities acquired through exposure to music with or without the support of explicit training. The capacities derived from implicit exposure achieve very high levels of sophistication, and enable untrained listeners to respond to music as trained listeners do (Bigand & Poulin-Charronnat, 2006). Nonetheless, explicit training can also alter the listener’s emotional experiences by priming the understanding of the musical structure in various ways and through an awareness of details in the music that impact emotions (even at the brain functioning level: e.g., Dellacherie, Roy, Hugueville, Peretz, & Samson, 2011).

Finally, *contextual factors* refer to the situational aspects of a particular music-listening experience that have an impact on the listener’s emotional experience. Central to these factors is the particular location of the performance or listening situation. This may be a concert hall, church, street, car, home, or a laboratory experiment, which has a direct impact on the auditory experience (e.g., the quality of sound depends on the acoustic response of the physical space; the music may be transmitted through loudspeakers, headphones, or without any technical support; the music may be heard without interruption or be disturbed by the sirens of an ambulance or the coughing of a concert visitor), but also a broader impact on the individual. Indeed, the specific nature of the listening situation, that is, whether it happens in the context of a particular event, such as a wedding, a funeral, or a celebration, may involve different goals and attitudes and even the adoption of specific behaviours and therefore may interplay with our emotional engagement with the music.

While some of these factors have been often studied by music psychologists (e.g., age, gender, musical background; see Scherer & Coutinho, 2013, for a detailed review of evidence related to each type of factor), others, particularly those related to context, have received very little attention and there is no systematic empirical study evaluating their effects on audiences’ affective experiences. Considering that many studies in music and emotion research are performed in a laboratory setting, isolated from the naturally occurring contexts in which emotional experiences with music most often happen, it is natural to assume that at least some aspects of the listening context will have an impact on the listener’s emotional experiences. As a consequence, it is important to start studying these issues by evaluating the *extent* to which contextual factors affect the listener’s emotional experiences; an issue that is pertinent to the question of whether laboratory settings provide an appropriate framework to study emotions in music. This is the first aim of this article—to examine empirically the similarities and differences between emotional experiences with pieces of music experienced in a live performance (ecological context) or in a laboratory study (experimental context). Clearly, we cannot hope to disentangle the many factors, and determine their relative effect, that vary between these two settings, and that are likely to modify the emotional responses of the listener, in particular the venue, the type of event, the knowledge, preferences and expectations of the participants. Our goal here is more modest. To orient the design of future studies attempting to control these factors experimentally, we wanted to study to what extent and in which direction the profiles of the emotional responses to a given musical performance will differ given the manifold differences between the two contexts.

In addition to the performance setting, we expected that the ability to see the movements and expressions of the performers would be a major factor affecting the emotional experiences of the audiences. Studies have shown quite conclusively, and somewhat surprisingly, that the visual perception of musicians’ performance on stage reliably influences both interpretations of expressive style and affective reactions to of music (Davidson, 1993; Krahé, Hahn, & Whitney, 2015; Platz & Kopiez, 2012; Thompson et al., 2005; Tsay, 2013; Vines et al., 2011). For instance, Thompson et al. (2005) and Vines at al. (2011), report that factors related to performer presence and expression (e.g., gestures, facial expressions, movements) affect the perception of music structure as well as the public’s affective experiences. This issue is of particular importance as listening to recorded music in a wide variety of everyday contexts has become a very frequent and widespread phenomenon in modern societies. This reality invites work on the differences in affective appeal between live and recorded music, including the issue of the immediacy and the modality (auditory, visual, or both) of the perception (Boltz, 2013; Finnäs, 2001; Kawase, 2014). Therefore, the second aim of this study was to study the impact of the perception modality of the listeners (audio-visual, audio-only, visual-only) on emotional experiences and compare these to the reaction profiles shown by the members of a concert audience. Whereas some of the earlier research in this area has mainly relied on dimensional ratings or basic emotion scales, we wanted to determine the emotional reactions by using a more fine-grained assessment instrument, a short version of the Geneva Emotional Music Scale (Zentner, Grandjean, & Scherer, 2008) developed to measure specific music-induced emotions.

In summary, our expectations were (1) that the emotional responses of the public attending the live performance would significantly differ from in those of the participants in the laboratory context; and (2) that being able to watch the interpretation of the performers should affect the emotion experienced reported by listeners in relation to the audio-only condition.

To address the issues described above, we created an empirical study in two contexts. In the first context, we focused on measuring the emotions experienced by volunteer members of an audience that attended a live music performance of a Lieder recital in a church setting (Context 1). In the second context, we focused on measuring the emotional reactions to the recording of the same musical pieces but in a controlled laboratory experiment with three different groups of participants that took place in a university lecture hall (Context 2). The three groups of participants were exposed to either the audio-video, the audio-only, or the video-only recording of the performance. As the danger of carry-over effects discourages the use of a repeated-exposure design, there were different participants in each context and condition.

With this experimental design the two specific research questions outlined in the introduction are investigated here. First, the emotional reaction profiles of the members of the audience in the live performance (natural context) are compared with those of participants recruited from the members of the public who were registered for a music festival, and were presented with a recorded version of the original live performance (laboratory context). Second, the effects of auditory and/or visual information on the emotional experiences of the three laboratory groups in Context 2 are evaluated. In relation to both goals, the differences between the various contexts and conditions in terms of the level of emotional convergence between participants (i.e., the degree to which similar emotions are reported by the members of a specific group) and the affective qualities of those emotional experiences are investigated. This exploratory study is meant to gauge the nature and extent of the differences in affective impact to be expected between live performances and recorded versions presented in the laboratory, allowing us to formulate more precise hypotheses for further work.

## Method

### Context 1

#### Design

**Figure 1.**
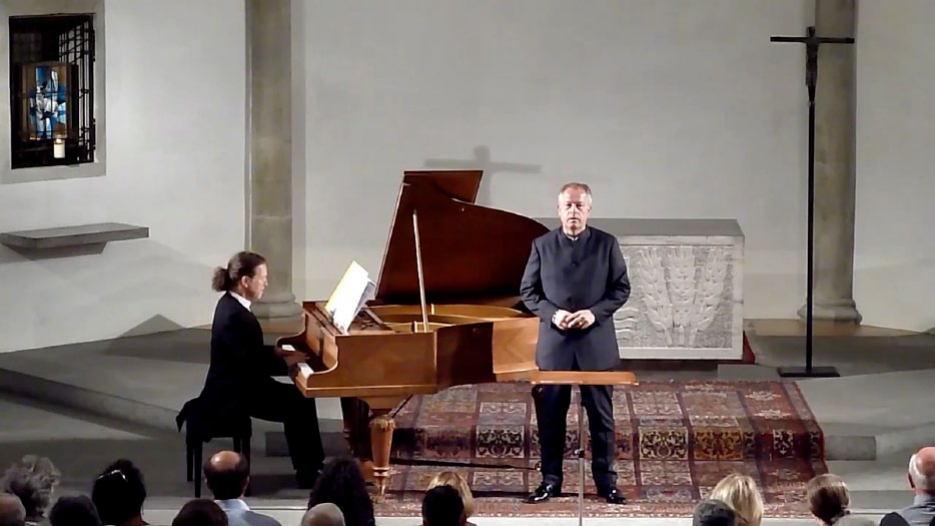
Still frame of the video-recording during the live performance.

This part of the study was conducted during a live performance (LIVE) at the Saint-Germain Church in Geneva, during the summer concert series (*Concerts d’Eté de Saint-Germain*) that takes place at this location every year. The music consisted of a Lieder programme (music for one singer and one piano) to poems by Johann Wolfgang von Goethe, with works mostly by Franz Schubert, but also by Hugo Wolf, Ludwig van Beethoven, Edvard Grieg, and Franz Liszt. To avoid disturbing the concentration of the audience and to obtain ratings immediately after each respective piece, we selected only three of the Lieder presented during the concert for our study (performed just before the intermission or the end of the concert): (1) Schubert’s *Die Liebe* (“Freudvoll und Leidvoll”) D210, (2) Wolf’s *Ganymed*, and (3) Liszt’s *Der du von dem Himmel bist* (first version). The performers were renowned tenor Christoph Prégardien and pianist Michael Gees. The entire performance was recorded using an HD video camera and a professional stereo microphone. The video camera and microphone were placed in front of the performers (in a position that did not disturb the view of the audience). The image captured included both the pianist and the singer and very little information about the physical context and surrounding environment, as shown in Figure 1. Both video and audio recordings were later processed to extract the sections corresponding to the stimuli used in our studies. There were no changes in the positions of the recording devices during the performance and no editing was performed.

#### Participants

Members of the audience were recruited before the concert started as they entered the venue. Participation was voluntary and there was no compensation. Participants who agreed to take part were given a questionnaire and a pencil. In the instructions, participants were asked to rate the emotions that they felt while listening to the three aforementioned pieces immediately after they were performed (in the intermission, or at the end of the concert). A total of 26 audience members (17 female, 3 male, 6 unknown), 26–79 years of age (*M* = 53, *SD* = 17) returned completed questionnaires at the end of the concert. The ages and genders of six participants were missing from the questionnaires. Additionally, nine ratings across all scales, pieces and participants (936 in total: 12 scales x 3 pieces x 26 participants) were missing. Missing values were not replaced. All members of the audience had access to a concert programme that included the schedule of the performance as well as the lyrics of all songs. No details about the emotional character of the music were included in the booklet.

#### Instruments and procedure

A revised short version of the Geneva Emotional Music Scale (GEMS; Zentner et al., 2008) was used. This instrument was expressly created for measuring musically induced emotions, comprising a set of feelings that are often reported while listening to music. The scale consists of 28 terms describing 12 classes of feelings of emotions, as reported by Coutinho and Scherer (2012) which are shown in Table 1. Participants had to rate how intensely they felt the emotion classes described by the items indicated in the table, using a five-point Likert scale ranging from 1 (*not at all*) to 5 (*very much*). Intermediate labels were as follows: *somewhat* (2), *moderately* (3), and *quite a lot* (4).

**Table 1. table1-0305735616670496:** Adapted version of the Geneva Emotional Music Scale used in our study.

Feeling class	Feeling items
Wonder	Filled with wonder, enchanted
Transcendence	Feelings of transcendence, awe, the sublime
Tenderness	Feelings of tenderness, love
Nostalgia	Nostalgic, melancholic
Peacefulness	Calm, relaxed, serene
Power	Feelings of power, triumph
Joyful activation	Joyful, lively
Tension	Tense, nervous
Sadness	Sad, sorrowful, depressed
Aesthetic feelings	Feelings of harmony, clarity
Epistemic feelings	Feelings of interest, discovery
Boredom	Bored, weary

At the end of the entire performance, participants were also asked to rate to what extent certain musical and non-musical determinants had had an impact on their emotional experiences. The list of potential determinants is shown in Table 2. Participants used a discrete scale ranging from 0 (*not at all important*) to 6 (*extremely important*) to provide their ratings.

**Table 2.** List of musical and non-musical factors in the listening experience potentially affecting listeners’ ratings of emotions felt.

Label	Determinant
Structure	Music structure, as written by the composer (i.e., tonality, intervals, melody)
Sound	Specific acoustic characteristics (e.g., the timbre of an instrument)
Interpretation	The interpretation of the performer(s)
Lyrics	The verbal content of the lyrics
Context	Contextual factors (e.g., the venue, other people)
Mood	The listener’s mood during the performance

### Context 2

All three conditions of this part of the study were conducted in a meeting room (approximately 100 m^2^ with 30 seats) at the University of Geneva. Participants were recruited via email from members of the public registered for a music festival and were asked to attend a recorded version of the three pieces selected for Context 1. Participants in Context 2 were allocated randomly to one of three groups: those who saw and heard the audio-visual recording of the stimuli (AV condition; 14 female, 2 male; age: *M* = 28, *SD* = 10, range = 20–53 years); those who listened to the audio-only version (AO condition; 17 female, 3 male; age: *M* = 28, *SD* = 12, range = 18–69 years); and those who saw the video-only version (VO condition; 10 female, 6 male; age: *M* = 26, *SD* = 6, range = 19–41 years). The sound (conditions AV and AO) was played through loudspeakers and the video (conditions AV and VO) was projected onto a screen at the front of the room. Participants sat side by side and in three rows (similar to a concert venue set-up) facing the projection screen and loudspeakers.

#### Instruments and procedure

In all conditions of Context 2, participants received the same rating sheet as used in the live performance. The only exception was the VO group: these participants did not complete the determinants questionnaire (see Table 2) because it was necessary to listen to the audio presentation to answer most of the questions.

## Results

**Figure 2.**
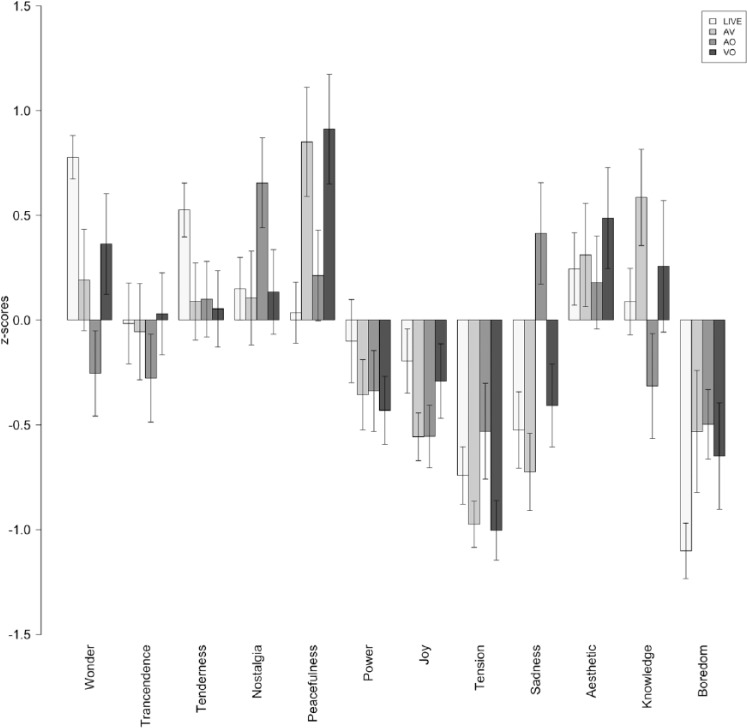
Piece 1: Ratings of felt emotions. LIVE = live performance; AV = audio-visual condition; AO = audio-only condition; VO = video-only condition.

**Figure 3.**
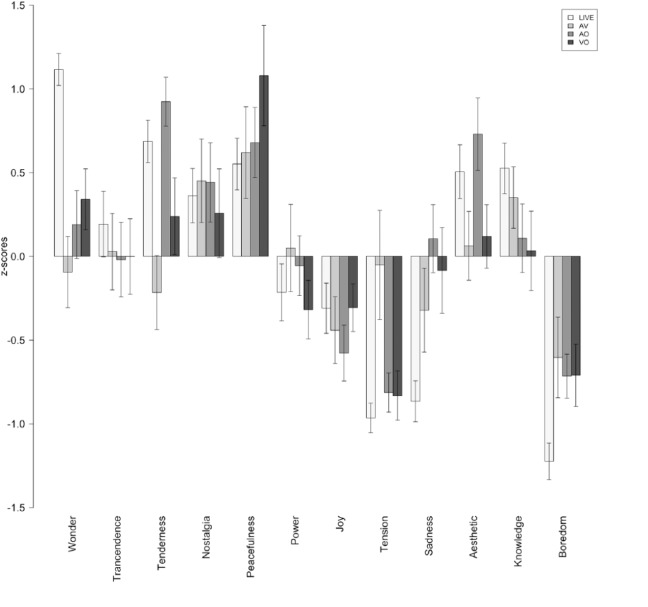
Piece 2: Ratings of felt emotions. LIVE = live performance; AV = audio-visual condition; AO = audio-only condition; VO = video-only condition.

**Figure 4.**
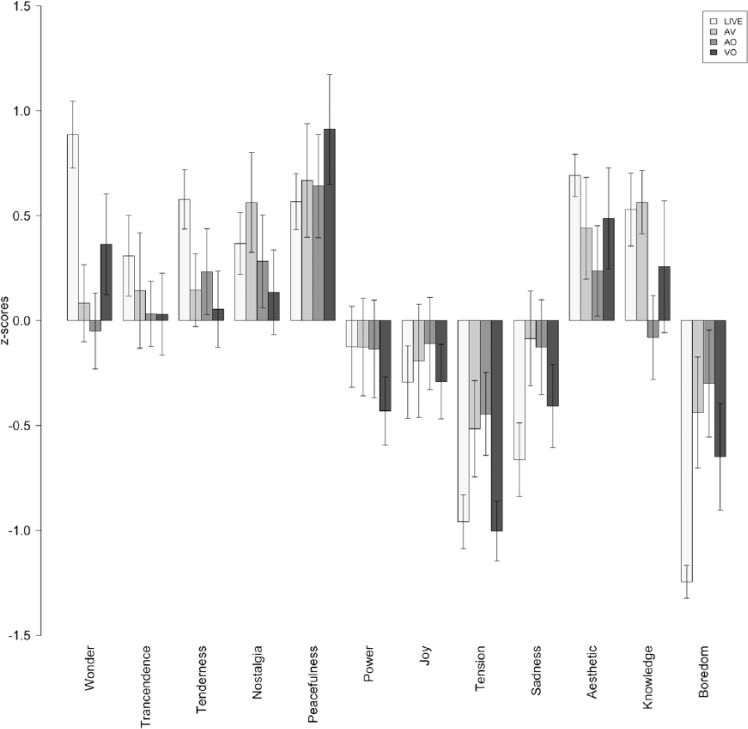
Piece 3: Ratings of felt emotions. LIVE = live performance; AV = audio-visual condition; AO = audio-only condition; VO = video-only condition.

**Figure 5.**
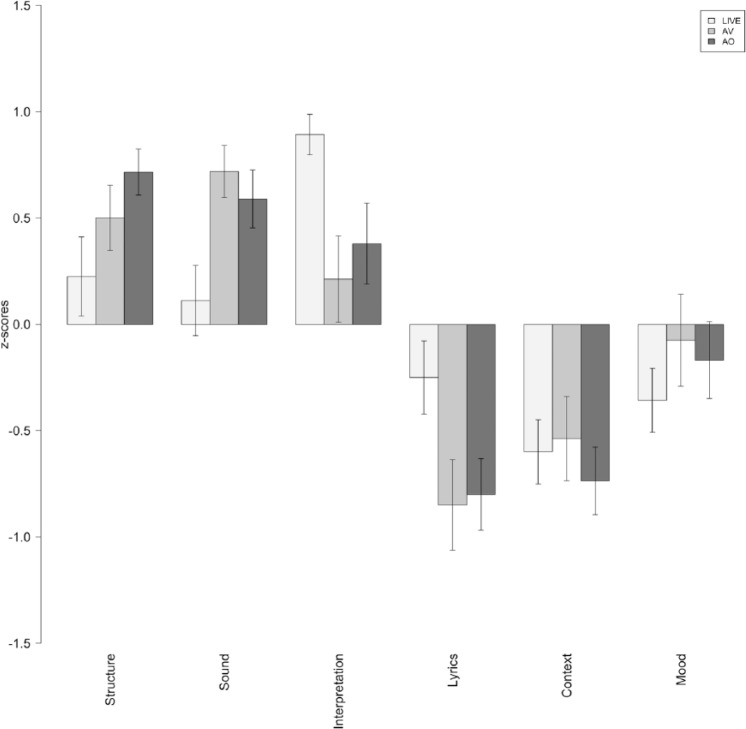
Importance given by participants in each experimental condition to the various factors of the listening context (determinants) with a potential effect on their emotional experiences. LIVE = live performance; AV = audio-visual condition; AO = audio-only condition; VO = video-only condition.

The raw ratings reported by the participants (emotion and determinants) were converted to standard scores (z-scores) in order to eliminate inter-individual response biases. Figures 2 to 4 show the average z-scores for participants in each experimental condition. Figure 5 shows the average ratings of the importance given by listeners to different factors of the listening context (determinants) affecting their emotional experiences.

In what follows, we will first evaluate the level of convergence across individuals, that is, the extent to which participants consistently experienced similar emotions in each condition. Then, we will compare the emotional profiles obtained for the various pieces and experimental conditions in order to evaluate the qualities of the emotions induced in the listeners of the various groups. Finally, we will describe how various factors related to the listening context (determinants) impacted the emotions experienced by the various groups of participants.

### Level of convergence between listeners

We used the Intraclass Correlation Coefficient (ICC; Shrout & Fleiss, 1979) to measure the consistency of participants’ ratings, and consequently the degree of emotional convergence (i.e., the extent to which listeners reliably felt the same emotions while listening to each music piece) on a per-group basis. In particular, we computed ICC(2,k) as described by Shrout and Fleiss (1979), which estimates the absolute agreement between participants for k ratings (in our case 3 pieces x 23 scales = 36 ratings). As can be seen in Table 3, results indicate an excellent (> .90) level of convergence among participants in the LIVE condition (ICC(2,k) = .94, *F* = 18, *p* < .001, lower bound = .90, upper bound = .97), which is higher than in any other condition. The level of convergence was, in the AV and AO groups respectively, .72 (*F* = 4.1, *p* < .001, lower bound = .57, upper bound = .84) and .74 (*F* = 4.2, *p* < .001, lower bound = .60, upper bound = .85), and .80 (*F* = 6.1, *p* < .001, lower bound = .69, upper bound = .89) in the VO condition.

**Table 3.** Inter-participant convergence (Intraclass Correlation Coefficient, ICC(2,k)) across all pieces for each experimental condition.

Condition	*N*	ICC(2,k)
LIVE	25	.94
AV	16	.72
AO	20	.74
VO	16	.80

*Note. N* indicates the number of participants in each condition. LIVE = live performance; AV = audio-visual condition; AO = audio-only condition; VO = video-only condition.

At first glance, these results suggest a tendency for a higher level of convergence (i.e., reporting similar feelings) in the live performance group relative to all other conditions. In order to substantiate this we used the test of equality of independent reliability coefficients proposed by Kim and Feldt (2008) to determine whether there were statistically significant differences between the levels of convergence in the various conditions (test implemented in the *cocron* library [Diedenhofen, 2013] of R [R Core Team, 2013]). Results show that the level of convergence between participants in the LIVE condition is significantly higher than in the AV (*p* < .001), AO (*p* = .001), and VO (*p* = .001) conditions. There were no statistically significant differences between AV, AO, and VO conditions (*p* > .05).

### Similarity between emotion profiles across and within conditions

To determine the similarity between the emotional experiences across experimental contexts and conditions, we calculated the linear profile correlation coefficients (*r*) between ratings (averaged across participants) on all scales and pieces (9 scales x 3 pieces = 36 observations) in the various conditions. This analysis serves to understand the global similarity across conditions, that is, the extent to which similar profiles of emotions were induced in all conditions. The results are shown in Table 4.

**Table 4.** Correlations between the profiles of emotions induced in each experimental condition (for all tests *p* < .001, *df* = 34).

	LIVE	AV	AO
**AV**	.65		
**AO**	.62	.50	
**VO**	**.78**	**.82**	.69

*Note.* LIVE = live performance; AV = audio-visual condition; AO = audio-only condition; VO = video-only condition. Values in bold indicate correlations above .75.

The first main observation is that all correlation coefficients are above .50 (*df* = 34 and *p* < .001 for all cases), which suggests that there is a considerable degree of similarity between the emotion profiles induced in the various groups of listeners. Nevertheless, the similarities vary considerably across conditions. In the laboratory studies, we obtained a correlation coefficient of .69 between the emotional responses of AO and VO groups, indicating a high degree of overlap between the emotions induced by the music (see also Figures 2 to 4) and those induced by the visual aspects of the performance. The correlations between the two unimodal groups (AO and VO) and the multimodal AV condition revealed a strong covariance between VO and AV (*r* = .82)—the highest of all the tests—and a moderate correlation between AO and AV (*r* = .50)—the lowest. These results indicate a strong contribution of the visual aspects of this performance on the emotion experienced by listeners, and their prevalence over audio cues. In relation to the comparison between the live performance and the three laboratory experiments, we found that the profile of emotions induced in the VO condition was the most similar to LIVE condition (*r* = .78), followed by AV (*r* = .65) and AO (*r* = .62). Once again, these values indicate that the visual aspects of the performance alone had a strong impact on the emotional experiences of listeners.

We turn now to the similarity between the emotion profiles induced in the listeners by the different pieces in each experimental condition. This analysis serves to assess how far the various pieces induced similar emotion profiles, and it was quantified by calculating the average of the linear correlation coefficients between each pair of pieces in each condition. The results obtained were .97 (LIVE), .82 (AV), .76 (AO) and .94 (VO), showing that the three pieces induced fairly similar emotion profiles in the participants. This is particularly evident in the LIVE and VO conditions, which seems to suggest that the performers visually communicated a stable set of emotions during the entire performance which were consistently induced in the public.

### Effects of contextual factors and modality of presentation

We now turn to the analyses of the effects of each experimental condition on the emotions experienced by the listeners. As, by necessity, the design was not completely randomised (the participants in the LIVE group were not drawn from the same population as the laboratory sample) and age and gender distributions were unbalanced, we decided to control for the age and gender of participants in order to mitigate the possible effects of confounding variance. For each emotion scale (E1–E12; dependent variables), data were analysed using a mixed-design analysis of covariance (ANCOVA) with a between-subjects factor of condition (LIVE, AV, AO, VO), a within-subjects factor of stimulus (Piece 1, Piece 2, Piece 3), and age and gender (dummy coded) as covariates. For those analyses where the sphericity assumption was violated (Mauchly’s test, *p* < .05), the degrees of freedom were adjusted by using the Greenhouse–Geisser correction.

The ANCOVAs revealed various main and interaction effects with medium or high effect sizes, and statistically significant at 5% significance level. These were further analysed by means of pairwise comparisons (corrected for multiple comparisons by using the Bonferroni adjustment). The effect sizes of the ANCOVAs *F*-tests were quantified with omega squared (ω^2^), and those of the pairwise comparisons with Cohen’s *d*. In the following paragraphs, we describe the main results separately for each feeling scale measure. Our focus is the main effects of condition and stimulus, as well as the interactions between both. The main effects of the covariates age and gender, and their interactions with stimulus are not the focus of this article. The detailed results of the 12 ANCOVAs are presented in [Table table1-0305735616670496] in the Table 1 online supplementary materials.

#### Wonder

There was a significant main effect of experimental condition on Wonder ratings after controlling for age and gender with a large effect size: *F*(3, 61) = 6.568, *p* < .001, ω^2^ = .195. Pairwise comparisons revealed that Wonder ratings were higher in the LIVE condition when compared to AV (*p* = .016, *d* = 0.703) and AO (*p* = .001, *d* = 0.859) conditions (medium and large effect sizes, respectively). Additionally, Wonder ratings were also significantly higher (with a medium effect size) in the VO condition compared to the AO condition (*p* = .046, *d* = 0.543), but not statistically different from the LIVE and AV conditions. There were no main effects of stimulus.

#### Tenderness

There were no main effects of condition or stimulus, but there was a significant interaction between both: *F*(5.215, 106.044) = 3.01, *p* = .013, ω^2^ = .088 (medium effect size). Pairwise comparisons revealed that ratings of Tenderness for Piece 2 were higher in the AO condition than AV (p < .001, *d* = 1.155) and VO (p = .043, *d* = 1.183), both with very large effect sizes.

#### Peacefulness

There was a main effect of condition on Peacefulness ratings: *F*(3, 63) = 2.84, *p* = .045, ω^2^ = .075 (medium effect size). Pairwise comparisons did not reveal significant differences amongst conditions at a 5% level, but there two contrasts yielded medium effects sizes—ratings of Peacefulness tended to be higher in the VO condition than in the LIVE (*p* = .089, *d* = .572) and AO conditions (*p* = .072, *d* = .523).

#### Power

We found a main effect of stimulus with a medium effect size: *F*(2, 63) = 8.65, *p* < .001, ω^2^ = .093. Pairwise comparisons did not yield any significant differences between pieces.

#### Tension

We found a significant interaction between condition and stimulus: *F*(6, 128) = 3.63, *p* = .002, ω^2^ = .102). Pairwise comparison showed that, for Piece 2, the ratings of Tension were significantly higher in the AV condition compared to the LIVE (*p* = .036, *d* = 1.216) and AO (*p* = .022, *d* = 1.123) conditions.

#### Sadness

We found a significant main effect of condition with a medium effect size: *F*(3, 63) = 3.23, *p* = .028, ω^2^ = .088. Pairwise comparisons revealed that Sadness was higher in the AO condition in relation to the LIVE condition (*p* = .046, *d* = .581). This effect was accompanied by an interaction with stimulus (*F*(6, 126) = 2.39, *p* = .032, ω^2^ = .057): Sadness ratings for Piece 1 in the AO condition were significantly higher than all other conditions (LIVE: *p* = .036, *d* = 1.230; AV: *p* = .002, *d* = 1.494; VO: *p* = .019, *d* = 1. 270).

#### Aesthetic feelings

An interaction between stimulus and condition was found (*F*(6, 126) = 2.64, *p* = .019, ω^2^ = .0.66), but pairwise comparison of adjusted means resulted in differences with small effect sizes and not statistically significant at the 5% level.

#### Epistemic feelings

We found a main effect of condition with a medium effect size (*F*(3, 64) = 2.87, *p* = .043, ω^2^ = .075). Pairwise comparisons did not reveal any statistically significant differences between conditions.

#### Boredom

Finally, we found a main effect of condition with a medium/high effect size on Boredom ratings: *F*(3, 64) = 3.67, *p* = .017, ω^2^ = .105. Pairwise comparisons revealed that Boredom was significantly lower in the LIVE conditions when compared to AV (*p* = .035, *d* = .634) and AO (*p* = .019, *d* = .646) conditions (large effect sizes).

### Determinants

To explore the role of the musical and non-musical determinants described in Table 2 (Structure, Sound, Interpretation, Lyrics, Context, Mood) on the emotions reported by participants in each experimental condition (except VO condition because most determinants refer to the music, and therefore the questionnaire was not administered), we conducted multiple one-way ANOVAs with condition (LIVE, AV, AO) as the between-subjects factor.

There were significant main effects (with medium and large effect sizes) of Sound, *F*(2, 57) = 4.69, *p* = .013, ω^2^ = .111; Interpretation, *F*(2, 58) = 5.59, *p* = .006, ω^2^ = .133; and Lyrics, *F*(2, 58) = 3.60, *p* = .034, ω^2^ = .080. Tukey’s HSD post-hoc analysis revealed that specific acoustic characteristics of the music (Sound) were significantly more important in the AV condition than in the LIVE condition (*p* = .002, *d* = .892) and that the performers’ interpretation (Interpretation) was rated as significantly more important in the LIVE condition compared with the AV (*p* = .009, *d* = .976) and AO (*p* = .046, *d* = .737) conditions. There were no significant differences across conditions in relation to the Lyrics determinant (*p* > .05), but the analysis of effect sizes revealed that lyrics were more important in the live condition compared to AV (*p* = .066, *d* = .725) and AO (*p* = .079, *d* = .665). Taken together, these findings suggest that in the laboratory condition, participants tended to rate the role of the music itself as a more important determinant of their emotional responses, whereas in the live performance, the interpretation by the performers was judged as relatively more important. Additionally, the lyrics were rated as more important in the LIVE condition, which may be because the people attending the concert had a particular interest in the pieces being performed (they chose to attend the concert) and had access to the lyrics (poems) through the concert programme.

## Discussion

In this study, we evaluated the role of contextual factors in listening to music (venue and occasion) and of modalities of presentation (audio and/or video) in the emotions experienced by various groups of participants. Our main expectations were (1) that the emotional responses of the public attending the live performance would significantly differ from in those of the participants in the laboratory conditions; and (2) that being able to watch the interpretation of the performers would affect the emotion experienced reported by listeners in relation to the audio-only condition.

We started by showing that people’s emotional responses in the live performance context are extremely consistent, and that higher levels of emotional convergence are achieved in the live performance compared to the laboratory conditions. These results indicate that the level of convergence between participants is affected by the particular context of the listening experience. Possible reasons for these differences are the presence of the musicians, the physical venue and the type of event/occasion. Indeed, the live performance occurred in the context of an annual concert series that attracts a particular type of public. Additionally, the fact that those who attended the live performance chose to be present, and most probably had particular expectations regarding the performers and the repertoire, may have led to higher levels of appreciation, motivation, and attention to the performance. Nevertheless, since listeners’ backgrounds could not be assessed in this study we cannot empirically confirm this impression.

The comparison between the various groups of listeners showed a considerable degree of similarity between the emotional experience profiles reported by the listeners in the various groups. Nevertheless, the similarities varied considerably across conditions. In the laboratory studies, we obtained a strong correlation between the emotional responses of audio-only and video-only groups, indicating a high degree of overlap between the emotions induced by the music, and those induced by the visual aspects of the performance (see also Figures 2 to 4). The correlations between the two unimodal groups (audio- and video-only) and the multimodal audio-video condition revealed a strong contribution of the visual aspects of the performance on the emotions experienced by listeners, and their apparent prevalence over audio cues. Furthermore, the profile of emotions induced in the VO condition was the most similar to LIVE condition (followed by AV and AO). Once again, these values indicate that the visual aspects of the performance alone played a major role in producing emotional experiences via music. A possible explanation for the closer similarity between the emotion profiles induced in the live and the video-only conditions is the fact that the participants in the former condition were more influenced by the performers’ interpretation and behaviour than by the music itself. This view is supported by two observations: (1) the acoustic characteristics of the music was significantly less determinant of the emotional experiences in the live condition compared to the audio-video condition; and (2) the performers’ interpretation was rated as significantly more important in the live condition compared with the audio-video. These observations are consistent with previous research (e.g., Thompson et al., 2005; Vines at al., 2011), which has identified factors related to performer presence and expression (e.g., gestures, facial expressions, movements, overall appearance) as potential modulators of the public affective experiences. Still, these results are quite surprising in light of the fact that music is considered to be mainly an auditory phenomenon and the widespread acceptance and frequent use of audio-only recordings. Nevertheless, they are congruent with previous research by Tsay (2013), who has shown that people may rely primarily on visual information for making judgments about a music performance, and the meta-analysis by Platz and Kopiez (2012) on audio-visual presentation that demonstrates that the visual component is an important factor in the communication of meaning (including expressive meaning).

In another correlational analysis, we focused on the similarity between the emotions induced in the listeners by the different pieces in each experimental condition. The results showed that the three pieces induced fairly similar emotion profiles in the participants. This is particularly evident in the live and video-only conditions, which seems to suggest that the performers visually communicated a stable set of emotions during the entire performance, which were consistently induced in the public (as shown by the level of emotional convergence in both these conditions). This seems to be corroborated by the fact that the lowest correlation was found in the audio-only condition, which also indicates a higher differentiation in the emotions induced in the listeners for the various pieces in relation to the presentations that included the visual component. Still, there was a high correlation between the emotions induced by the different pieces in the audio-only condition, which seems plausible given that all Lieder in the performance are part of the romantic period in the Classical repertoire. It may be necessary to use more fine-grained instruments and a highly knowledgeable audience to detect Lied-specific differences.

The specific effects of experimental condition and piece (controlling for age and gender) in the emotional experiences of the various audiences were further explored through an analysis of variance. Looking first at the effects of experimental condition, we found various significant differences amongst groups related to the experience of particular emotions: Wonder, Sadness and Boredom. Wonder ratings were higher in the live performance than in the audio-video and audio-only conditions, and those in video-only condition were also higher than those in the audio-only condition. Boredom ratings were lower in the live condition when compared to the audio-video and audio-only laboratory conditions. Finally, Sadness ratings were higher in the audio-only condition than in the live performance. Perhaps surprisingly, there were no significant differences between live and video-only conditions as a result of the experimental condition, which may again attest to the relative power of the visual cues as highlighted by the correlation analysis.

In relation to Wonder and Boredom, the lack of difference between live and video-only conditions suggests a central role of visual information (especially the performers’ behaviour, given that in the video-only condition very little information about the venue is conveyed) in the emotional responses of the live performance group, to some extent independently of the music (given that Wonder ratings in the audio-only condition are significantly lower than the video-only and live conditions). This is congruent with the similarity between emotion profiles induced in the live and video-only condition (as discussed above) and with our analysis of the factors reported by listeners as being determinant elements in their emotional experiences—listeners in the live performance attributed more of the emotional effect to the interpretation of the performer, whereas in the laboratory, participants tended to rate the music as a more important determinant of their emotional responses. One central and plausible source of explanation for these results is the level of listeners’ engagement with the performance and performers, including the level of empathy. The participants in the video-only condition were only exposed to the visual cues and therefore their only source of information is the performers’ behaviours, but the audience members in the live performance were also exposed to the music itself and still their experiences are similar to those in the video-only group. Arguably the members of the public in the live performance were more familiar with the reputation of the singer and the pianist (given that most members of the audience actively chose to attend that particular concert), and thus their emotional experiences may have been driven more by the performers’ reputation and behaviour than by the music (as is also corroborated by the importance attributed by the live performance group to the performers’ interpretation). In contrast, the participants in the laboratory sessions were unlikely to be familiar with the performers’ background and reputation. This would also explain the low levels of Boredom in the live performance compared with those in the laboratory, suggesting higher levels of engagement with the performance—possibly in part due to the prestige effect of the famous performers. This explanation is supported by previous research showing that music-induced emotions (including physiological arousal) are affected by manipulations of empathy towards the performers (Miu & Balteş, 2012). Furthermore, the results also support the idea that live events are intrinsically more interesting, since, although the live concert was longer than the laboratory sessions, ratings of Boredom were lower.

Concerning Sadness, our results showed that ratings were significantly higher in the audio-only condition compared to the live performance; an effect that was accompanied by an interaction with piece, in such way that for Piece 2 higher ratings of Sadness were reported in the audio-only condition in relation to all other conditions. This indicates that Sadness was induced by the music itself but also that that the experience of this emotion may be partially lowered by the introduction of visual cues showing the performers’ expressive behaviour. This would fit findings in the emotion expression literature, suggesting that facial cues are better indicators of valence whereas voice cues communicate arousal and power. Once more this suggests that visual cues play a powerful role in the induction of emotion through music to the point of partially masking the emotions conveyed by the music.

As to the main effects of piece, we found that only the ratings of Power differed significantly from piece to piece but pairwise comparisons did not reveal any significant differences. This suggests that the three pieces induced similar emotional experiences. However, as in the case of Sadness, there were various interactions between stimulus and condition in the ratings of Tenderness, Tension and Aesthetic feelings, indicating a higher level of differentiation between pieces. Pairwise comparisons revealed significant differences regarding Tenderness and Tension. Starting with the ratings of Tenderness, we found that, for Piece 2, they were significantly higher in the audio-only condition compared to the audio-video and video-only conditions (but not live). On one hand, this suggests that Tenderness is a particularly important emotion in this piece, which indeed seems to be the case, given that, among other things, Goethe’s poem *Ganymed* orbits around the union between man and divine nature and expresses feelings of beauty, love, and tenderness. On the other hand, these results can indicate that Tenderness was not communicated visually. This interpretation might also offer an alternative explanation for the ratings of Sadness for Piece 1, which were also significantly higher in the audio-only condition compared to all other conditions. This might again reflect the fact that the voice is a privileged medium to communicate low arousal and low power. Once more, this indicates that the music conveyed a particular emotion that may have been masked by the interaction with visual cues. Finally, the ratings for Tension were significantly higher in the audio-video condition compared to the live performance and audio-only condition. This finding might be due to the fact that facial and gestural signs of tension by the interpreter might be more easily identifiable in the video image rather than from the back rows of the church.

## Conclusion

As mentioned at the outset, the process of emotion induction through music presents a formidable challenge for empirical research. This study presents some evidence that factors related to the visual aspects of the performance can have a determinant impact on the emotional experiences of audiences. Therefore, it seems fundamental to consider carefully the modalities of presentation of particular music performances when investigating emotional responses to music both in laboratory studies and live performances. Furthermore, the multifaceted interactions between audio and visual cues suggest that the experience of musical emotions is far more complex than is often assumed and that audio-visual integration should be carefully and systematically evaluated (see also Finnäs, 2001 and Platz & Kopiez, 2012). Perhaps because we live in the era of digital music, music experiences are often considered as purely auditory; nonetheless, it is fundamentally important to notice that the cause of this is the proliferation of recorded music, a recent technological advance, which has detached the aural and visual dimensions of music. A full understanding of the emotional power of music requires systematic investigation of the interactive effects of visual and auditory signals, particularly at a moment in time in which the visual cues accompanying music are no longer confined to live performers’ behaviours and appearance in the form of nonverbal information, but span a wide range of visual media, often detached from real-life experience (Thompson et al., 2005).

As some of the issues in this article were being addressed for the first time, there was little guidance for the design and the procedures used in the studies reported here. In consequence, several limitations of the present study need to be acknowledged, which will allow us to formulate more precise hypotheses for further work. The first limitation relates to the number of participants in each condition. In future work, a larger number of participants should be recruited to increase the power of the statistical tests and to facilitate the emergence of other relevant effects. The second limitation pertains to the conditions in the experimental design. It would be ideal to have audio-only and video-only conditions also in the live performance in order to determine specific effects of the location and the performers’ expression. A possible solution would be to provide blindfolds and earplugs to experimental groups of participants in the audience (although this might be difficult to achieve while keeping the intrinsic motivation of typical listeners intact). A third limitation is the absence of appropriate control over the participants’ backgrounds. It is highly plausible to assume that some of the differences found between the live performance condition and the laboratory conditions are due to listener-related factors, such as those presented in the introduction and mentioned throughout the discussion. Although the effects of age and gender have been partialed out, one would expect that the public present at the live performance, having freely chosen to attend the concert, might have had very different expectations and background knowledge about the type of music being played, as well as empathy towards the performers. Some recently developed instruments, namely the PROMS (Law & Zentner, 2012), which assesses music abilities objectively, and the MUSE (Chin & Rickard, 2012), which measures engagement with music, are potentially useful tools. Nevertheless, the amount of time and effort required for the administration of these instruments to normal audiences is quite large, which creates a barrier to its applications in ecological contexts (such as concerts). A much-needed methodological requirement for future research is to develop adequate background questionnaires that capture individual aspects relevant to the induction of emotions through music (such as age, musical training, personality traits and musical preferences). Another desideratum for future research is the development of experimental designs that allow comparing the effects of vocal and instrumental music, in particular with respect to the verbal content of the texts interpreted by singers. Another issue of interest is to examine in greater detail the fact that reactions to the video-only presentation were rather similar to those in the conditions that involved actually listening to the music. In fact, one of the reviewers pointed out that the video image conveyed information about the church setting of the live concert which might have resulted in ambiguous or conflicted context cues. Another important aspect to consider (and to measure) would be social or normative expectations that govern the listening situation of specific genres of music and thereby might produce convergent affective reactions (see Egermann, Kopiez, & Altenmüller, 2013).

This study explored the scope and the nature of differences in affective impact of a live music performance and the presentation of different recorded versions of the same pieces in the laboratory. As expected, we have confirmed the existence of significant differences in the type and strength of the impact. However, the results also show considerable similarities in affective responses, suggesting that while laboratory research on musical induction of emotion cannot hope to capture the richness of experience found in live performances, this approach still allows the systematic study of some fundamental mechanisms of emotion induction through music. This provides strong encouragement of further experimental work on the emotional impact of music and the role of different types of music-structural, performance and interpretation, context and listener factors. It will be the task of further work in this area to disentangle in a more fine-grained fashion the role of the different influence factors that were necessarily confounded in the present work. The challenges faced by this type of research can only be addressed by designing future studies on the basis of a systematic exploration of these factors and their interactions (see Scherer & Coutinho, 2013). Further work on these issues might provide a sufficiently solid basis to derive specific hypotheses from the theoretical framework proposed in the introduction. This would allow engagement in hypothesis-testing approaches rather than the exploratory stance we have had to take in this initial endeavour. In fact, the very notion of live versus media-conveyed performances deserves further theoretical attention. Apart from the difference in setting—concert versus laboratory—what is it that constitutes the uniqueness of the live presence of the performers—a sense of reality, spontaneity, unpredictability, credibility, increased empathy or still other factors? To advance on these questions, in future research it may be conceivable to combine field studies and laboratory experiments in appropriate settings with captive audiences, for example at festivals or conventions. An additional desideratum is to strongly increase the degree of multidisciplinary collaboration in this type of experimental research on the emotional impact of music, bringing together the competences of researchers in different fields of music studies, emotion researchers, and experts in methodology.
